# A Multi-Omics Analysis of Bone Morphogenetic Protein 5 (*BMP5*) *mRNA* Expression and Clinical Prognostic Outcomes in Different Cancers Using Bioinformatics Approaches

**DOI:** 10.3390/biomedicines8020019

**Published:** 2020-01-21

**Authors:** Md. Adnan Karim, Abdus Samad, Utpal Kumar Adhikari, Md. Ashraful Kader, Md. Masnoon Kabir, Md. Aminul Islam, Md. Nazmul Hasan

**Affiliations:** 1 Department of Genetic Engineering and Biotechnology, Jashore University of Science & Technology, Jashore 7408, Bangladesh; 2 School of Medicine, Western Sydney University, Campbelltown, NSW 2560, Australia; 3 Laboratory Science & Service Division (LSSD), International Centre for Diarrhoeal Disease Research, Dhaka 1213, Bangladesh

**Keywords:** *BMP5*, *BMP5* mutations, cancer prognostic biomarkers, cancer progression, multi-omics analysis, protein–protein interaction, bioinformatics

## Abstract

Cumulative studies have provided controversial evidence for the prognostic values of bone morphogenetic protein 5 (*BMP5*) in different types of cancers such as colon, breast, lung, bladder, and ovarian cancer. To address the inconsistent correlation of *BMP5* expression with patient survival and molecular function of *BMP5* in relation to cancer progression, we performed a systematic study to determine whether *BMP5* could be used as a prognostic marker in human cancers. *BMP5* expression and prognostic values were assessed using different bioinformatics tools such as ONCOMINE, GENT, TCGA, GEPIA, UALCAN, PrognoScan, PROGgene V2 server, and Kaplan–Meier Plotter. In addition, we used cBioPortal database for the identification and analysis of *BMP5* mutations, copy number alterations, altered expression, and protein–protein interaction (PPI). We found that *BMP5* is frequently down-regulated in our queried cancer types. Use of prognostic analysis showed negative association of *BMP5* down-regulation with four types of cancer except for ovarian cancer. The highest mutation was found in the R321*/Q amino acid of *BMP5* corresponding to colorectal and breast cancer whereas the alteration frequency was higher in lung squamous carcinoma datasets (>4%). In PPI analysis, we found 31 protein partners of *BMP5,* among which 11 showed significant co-expression (*p*-value < 0.001, log odds ratio > 1). Pathway analysis of differentially co-expressed genes with *BMP5* in breast, lung, colon, bladder and ovarian cancers revealed the *BMP5*-correlated pathways. Collectively, this data-driven study demonstrates the correlation of *BMP5* expression with patient survival and identifies the involvement of *BMP5* pathways that may serve as targets of a novel biomarker for various types of cancers in human.

## 1. Introduction

Cancer is one of the leading causes of premature death [1,2]. According to the Cancer Statistics for 2019, the number of new cancer cases yearly in the United States alone for all sites combined was 1,762,450 with an estimated death totaled 606,880 [3]. Furthermore, the number of cancer patient is increasing due to population and aging [2]. One of the most dominant causes of oncogenesis is the accumulation of gene alterations which has a positive correlation to the prognosis of cancer patients. Cancer can be detected early by using diagnostic cancer markers that identify differentially expressed genes associated with the survival of cancer patients. Moreover, these diagnostic biomarkers can be exploited as therapeutic agents by successfully understanding their mechanism of alteration.

Bone morphogenetic protein 5 (*BMP5*) is a member of the transforming growth factor-β (TGF-β) superfamily of signaling cytokines [4], which is located on the chromosome 2 in mice chromosome 20 in humans [5]. It acts through either autocrine or paracrine mechanism by binding to cell surface receptors and initiate a sequence of downstream events that have effects on various cell types. *BMP5* is known for their ability to induce bone and cartilage development, differentiate osteoprogenitor mesenchymal cells and up-regulation of osteoblastic features by their direct and indirect influence over cytokines and growth factors [6]. Mutation in *BMP5* is associated with a wide range of skeletal defects such as reduction in the long bone width and the size of the vertebral processes as well as the reduction of lower body mass [7,8]. It has been reported that BMP, together with its subtype *BMP5*, is involved in the initiation of several types of cancers such as colon, lung, breast, bladder, and ovarian cancer [9,10,11,12,13,14,15,16]. Previous studies on colorectal cancer showed a significant correlation between *BMP5* down-expression and mutation and the prognostic value of colorectal cancer (CRC), triggering the initiation and development of tumors [12,14,15]. One study reported that 13 cases of *BMP5* mutation across seven cancers where gastrointestinal cancers (GICs) were the most influenced by recurrent hotspot mutations [13]. Likewise down-regulation of *BMP5* has also been reported in other types of cancer such as adrenocortical carcinoma [9]. In contrast, the over-expression of *BMP5* was observed in lung adenocarcinoma (LUAD) [13,17], and breast cancer [18,19]. However, later studies showed a positive correlation of *BMP5* down-regulation with lower relapse-free survival in breast cancer patients and can be used as a therapeutic strategy combined with TGFβ1 to reduce cellular proliferation [20,21]. Down-regulation of *BMP5* has also been reported in 6 out of 10 lung squamous cell carcinomas (LUSC) [22]. These findings suggest that *BMP5* has an essential role in various cancer progressions.

To study the expression and evaluation of *BMP5* as a potential prognostic value for the treatment of various cancers, we systematically analyzed the *BMP5* expression and its clinical outcomes in certain cancers. Numerous expression and patient survival datasets were used, which are available on various recognized online platforms. We also investigated the genes co-altered with *BMP5* in relation to the five cancer types with high *BMP5* expression. Therefore, these systemic analyses eventually determine whether *BMP5* expression can be used as a biomarker for the prognosis of human cancer.

## 2. Materials and Methods

### 2.1. Analysis of mRNA Expression Levels in Different Cancers

Analysis of mRNA expression was carried our using ONCOMINE (https://www.ONCOMINE.org/resource/login.html) [23,24]. The threshold for analyzing this gene was as follows; *p*-value: 1 × 10^−4^, fold change: 2, gene ranking: 10%; analysis type: cancer vs. normal, and data type: mRNA. The parameters were kept the same for all cancer types.

### 2.2. Exploration of Gene Expression Pattern in Different Cancers

Gene expression across normal and tumor tissue (GENT) (http://gent2.appex.kr/gent2/) is a web-accessible resource where gene expression patterns between diverse human cancer and normal tissues are explored [25]. To obtain differential expression patterns of *BMP5* in normal vs. cancer tissues, we used a set of default parameter on data sets, samples, and probes.

### 2.3. Extensive Analysis of Gene Expression Data

UALCAN (http://ualcan.path.uab.edu) is a publicly available web tool to perform in-depth analyses of TCGA gene expression data [26]. It can analyze gene expression, promoter methylation, correlation, and prognosis. Using UALCAN database, we analyzed the expression pattern of *BMP5* mRNA.

### 2.4. Exploration of RNA Sequence Expression in Different Cancer

The gene expression profiling interactive analysis (GEPIA) is another interactive online platform that can explore RNA sequence expression between normal and cancer samples from TCGA and GTEx project [27]. We utilized GEPIA (http://gepia.cancer-pku.cn/) for differential expression analysis of *BMP5* in tumor vs normal tissue from various cancers. It can also perform other functional analysis, including patient survival and correlation analyses. KM-Express (http://ec2-52-201-246-161.compute-1.amazonaws.com/kmexpress/index.php) a platform known for identification and functional characterization of novel RNA biomarkers and targets in cancer survival and gene expression was used as well for this study [28].

### 2.5. Determination of Potential Cancer Biomarkers

PrognoScan (http://dna00.bio.kyutech.ac.jp/PrognoScan/) is an online resource that was used to determine potential tumor biomarkers and therapeutic targets [29]. This platform has an extensive collection of cancer microarray datasets to assess the relationship between gene expression and patient prognosis. The parameter was adjusted to a Cox *p* < 0.05.

### 2.6. Survival Analysis by PROGgene V2 Database

PROGgeneV2 (http://watson.compbio.iupui.edu/chirayu/proggene/database/?url=progge) is a web-based platform that used to study prognostic implications of genes in different types cancer [30]. We applied PROGgeneV2 to perform survival analysis on *BMP5* as a signature in various human cancers. The threshold was *p* < 0.05.

### 2.7. Identification of Expression, Mutation, and Functional Protein Partners

The cBioPortal for Cancer Genomics (http://cbioportal.org) is a widely used web platform for exploring, visualizing, and analyzing multidimensional cancer genomics datasets [31,32]. It contains various types of data, including mRNA expression, DNA copy number, DNA methylation, non-synonymous mutations, and limited de-identified clinical data. cBioPortal was applied to explore expression, mutations, and CNAs of *BMP5* with defined parameter settings in this study. Besides, the OncoPrint sub-tool of cBioPortal was also utilized to analyze the integrated status of mutations and CNAs for *BMP5* and their functional protein partners.

### 2.8. Analysis of Protein–Protein Interaction

We used both the GeneMANIA and STRING servers for the protein–protein interaction analysis. Herein, GeneMANIA (https://genemania.org/) is an online interface used for generating hypotheses about gene function, analyzing gene lists and prioritizing genes for functional assays [33]. It is an extensive database of functional association data, including protein and gene interactions, pathways, and co-expression. We applied this database to predict the protein–protein interactions using *BMP5* as queries. The interactions between genes are displayed in the form of a network where nodes symbolize genes and links represent networks. STRING (https://string-db.org/) is another web-based data mining platform dedicated to protein–protein interactions, both physical and functional associations [34]. We used this database to determine the functional protein partners of *BMP5*. Several factors, including text mining, experiments, databases, neighborhood, gene fusion, and co-expression, were considered as active protein–protein interaction sources.

### 2.9. Finding Co-Expressed Genes of BMP5 and Its Pathway Analysis

Positively and negatively co-expressed genes of *BMP5* were explored in TCGA dataset of five different types of cancer (breast, colon, lung, bladder and ovarian), using the R2: Genomics Analysis and Visualization Platform version 3.2.0 (https://hgserver1.amc.nl/), with the adjustment of Bonferroni test and cutoff *p*-value was selected as < 0.01. Thereafter, the common gene set was explored by drawing Venn diagrams, using co-expressed genes from five different cancers.

To explore pathways and gene ontology shared by *BMP5*-correlated genes, we used Protein Analysis Through Evolutionary Relationships (PANTHER) tool (http://pantherdb.org/) [35] and GOnet: Gene Ontology Analysis (https://tools.dice-database.org/GOnet/) [36]. Subsequently classified them based on their KEGG (Kyoto Encyclopedia of Genes and Genomes) pathway (*p* < 0.05).

### 2.10. Statistical Analysis

Bar and forest plots were drawn using GraphPad Prism version 6 (GraphPad Software, La Jolla, CA, USA). All results are displayed with *p*-values obtained from a log-rank test (Supplementary Tables S2 and S3). R (version 3.6.1) programming language was used for custom scripts. Custom scripts based on pheatmap function as available in the ‘gplots’ Bioconductor project (http://www.bioconductor.org/) was used for hierarchical clustering and heatmaps. The fold-change of gene expression for heatmap were obtained from ONCOMINE database (Supplementary Table S4).

## 3. Results

### 3.1. BMP5 mRNA Expression in Various Types of Cancer

**Figure 1:**
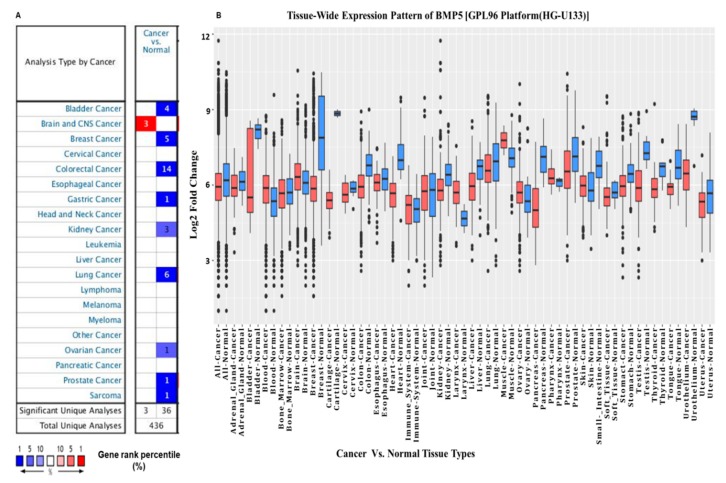
Bone morphogenetic protein 5 (*BMP5*) transcription level in different types of cancer derived from the ONCOMINE and Gene Expression across Normal and Tumor Tissue (GENT) databases. (**A**) This graphic was generated from the ONCOMINE database, indicating the number of datasets with statistically significant (*p* < 0.01) mRNA overexpression (red) or down-regulation (blue) of *BMP5* (different types of cancer vs. corresponding normal tissue). The threshold was designed with the following parameters: *p*-value of 1 × 10^−4^, fold change of 2, and gene ranking of 10%. (**B**) *BMP5* mRNA expression pattern in normal and tumor tissues. *BMP5* mRNA expression in various types of cancer was obtained from the GENT database. X-axis exhibits log2 fold change values of *BMP5* expression whereas, Y-axis shows the types of tissues *BMP5* were expressed. Boxes represent the median and the 25th, and 75th percentiles dots represent outliers. Red boxes represent tumor tissues; blue boxes represent normal tissues. Red and blue dashed lines represent the average value of all tumor and normal tissues, respectively.

We utilized databases such as ONCOMINE, GENT, The Cancer Genome Atlas (TCGA) through UALCAN, GEPIA, and KM-Express to investigate the differential expression pattern of *BMP5* in various cancer types and their counterparts. We queried with “*BMP5*” in ONCOMINE using the default threshold parameters (*p*-value: 1 × 10^−4^, fold change: 2, and Gene rank: 10%). In our study, total 436 unique analysis was reported by the ONCOMINE database among which 39 studies ranked *BMP5* within the top 10% showing significant (*p* < 0.05) statistical differences. Our obtained results revealed that *BMP5* expression was down-regulated in bladder, breast, colorectal, gastric, kidney, lung, ovarian, prostate cancer and sarcoma compared to the expression level in normal cases (Figure 1A) whereas up-regulation was only found to be in the central nervous system (CNS) cancer. We used the U133 platform of GENT database to obtain expression data of various cancer types compared to their normal counterparts by utilizing the microarray data profiled by Affymetrix platforms (see Supplementary Table S1). Herein, the results showed that *BMP5* expression was down-regulated in several cancer types including bladder, brain, breast, colon, kidney, liver, lung, ovary, pancreas, prostate, stomach, testis, and thyroid (Figure 1B). In contrast, up-regulation of *BMP5* was only reported in blood, head-neck, and skin cancer. According to the analysis using the GENT database, the average expression of *BMP5* was lower in cancer tissues than in the normal tissues. More to that, the analysis with the ONCOMINE and GENT database have shown that *BMP5* expression was apparently down-regulated in multiple cancer types, which includes bladder, breast, colorectal, lung, and ovary cancers. Therefore, we chose these respective cancer types (bladder, breast, colorectal, lung, and ovarian) for further systematic expression, prognosis, mutation, copy number alteration and protein–protein interaction analysis.

### 3.2. Expression Pattern of BMP5 mRNA in Different Cancer Types

**Figure 2:**
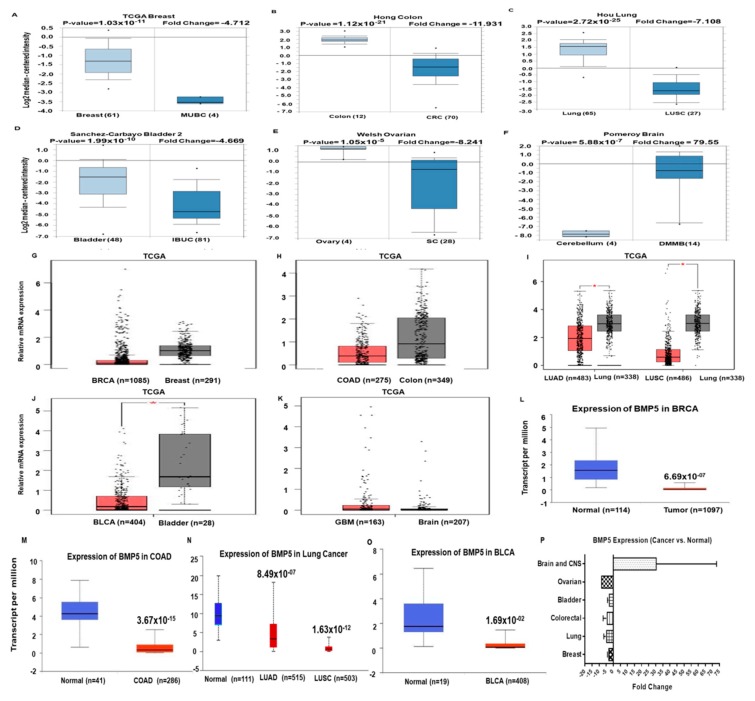
Expression pattern of *BMP5* was compared between cancer and tissue. (**A**–**F**) Fold-change of *BMP5* in six types of cancers was obtained from our analyses, shown as a box plot. The box plot comparing specific *BMP5* expression in normal (left plot) and cancer tissue (right plot) was derived from the ONCOMINE database. The above and below asterisk of the box represent maximum and minimum value, respectively. (**G**–**K**) *BMP5* expression data obtained from the TCGA database through GEPIA. The expression of *BMP5* in lung adenocarcinoma (LUAD) and lung squamous cell carcinoma (LUSC) were compared and profiled against normal lung tissue samples (see **I**). (**L**–**O**) *BMP5* gene expression in TCGA database. Box plots exhibiting the *BMP5* mRNA expression in the primary tumor (red plot), respectively and their normal (blue plot) tissues, using data from the TCGA database through UALCAN. *: *p* < 0.01. (**P**) The fold-change of *BMP5* in various types of cancers were compared in our analyses, as shown in Supplementary Table S1 and expressed as a bar plot.

We conducted cDNA microarray analysis by using the ONCOMINE database to explore the gene expression of *BMP5* in cancer types. The ONCOMINE database was queried for *BMP5* expression in cancer and normal tissues. Our analysis revealed that *BMP5* was down-regulated in breast, coloretal, lung, bladder and ovarian cancers, but was over-expressed only in brain and CNS cancers as compared to that in normal tissue (Figure 2A–F and Supplementary Table S1) [37,38,39,40,41,42,43,44,45,46,47,48,49,50,51,52]. Analysis of TCGA datasets through UALCAN and GEPIA database also exhibited similar *BMP5* expression in breast carcinomas, colorectal adenocarcinomas, lung squamous cell and adenocarcinomas, bladder cancer and glioblastomas (Figure 2G–O). Furthermore, the forest plot obtained from our statistical analysis shows an overall fold change pattern of *BMP5* expression in six different cancer types reported in Supplementary Table S2 (Figure 2P). Since the expression status of *BMP5* was confirmed in multiple databases, these results can be considered reliable.

### 3.3. Prognosis Analysis of BMP5 mRNA Expression in Cancer Patients

**Figure 3:**
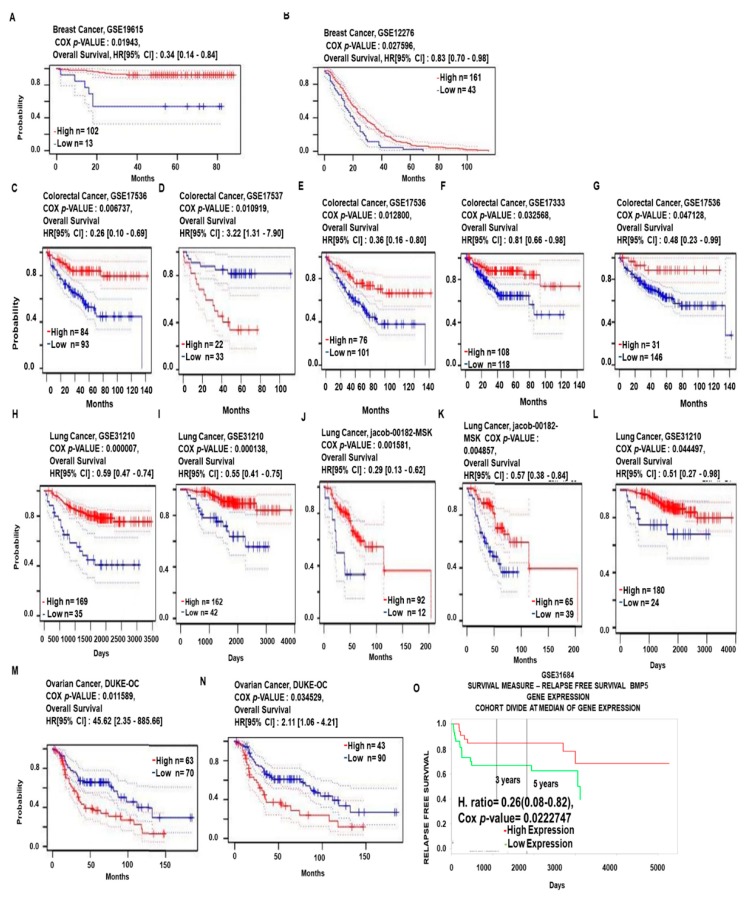
Relationship between *BMP5* expression and prognosis in various cancer patients. The survival curve comparing patients with high (red) and low (blue) expression in breast cancer (**A**,**B**), colorectal cancer (**C**–**G**), lung cancer (**H**–**L**), ovarian cancer (**M**,**N**) were retrieved from the PrognoScan Database. (**O**) Comparison of high (red) and low (green) *BMP5* expression in bladder cancer patients plotted from the PROGgene V2 database. Survival curve analysis was conducted using a threshold Cox *p*-value < 0.05. The dotted lines represent maximum and minimum values of the survival average.

We summarized the prognostic value of *BMP5* mRNA expression in various cancers by using the patient prognosis data from databases with significant Cox p-values (*p* <0.05). We used the PrognoScan database to analyze the association of *BMP5* expression with survival ratio of brain and CNS, breast, colorectal, lung and ovarian cancer patients. In breast cancer, GSE19615 and GSE12276 datasets showed that patients with lower *BMP5* expression (*n* = 13 and 43, respectively) had significantly lower overall survival compared to patients with higher *BMP5* expression (*n*= 102 and 161, respectively) (Figure 3A,B; and Supplementary Table S3). Similar results were also obtained in colorectal cancer datasets (Figure 3C–G; and Supplementary Table S3). In Figure 3C,E–G the GSE17536 and GSE17333 datasets exhibited that the patient group with low level of *BMP5* mRNA expression (*n* = 93, 101, 118 and 146, respectively) reported significantly poor overall survival compared to the high expression group (*n* = 84, 76, 108 and 31, respectively), whereas one alteration reported by dataset GSE17537 contradicted the association of *BMP5* low expression with overall survival of colorectal cancer patients (Figure 3D). Analysis of GSE31210 and Jacob-00182-MSK datasets of PrognoScan showed significantly (*p*-value) lower survival of lung cancer patients in the low *BMP5* mRNA expression group (*n* = 35, 42, 12, 39 and 24, respectively) compared to their higher expression counterparts (*n* = 169, 162, 92, 65 and 180, respectively; Figure 3H–L and Supplementary Table S3). High survival ratio was exhibited in low *BMP5* expression group (*n* = 70 and 90, respectively) of ovarian cancer compared to the high expression group (*n* = 63 and 43, respectively), according to the DUKE-OC dataset (Figure 3M,N and Supplementary Table S3). PROGgene V2 database was used to determine the prognosis of *BMP5* expression with bladder cancer (Figure 2O). In GSE31684 dataset, relapse-free survival was significantly low in bladder cancer patients with the low expression level of *BMP5* compared to their higher expression counterparts (Cox *p*-value: 0.0222747). These overall data-driven results suggest that apart from one alteration in colorectal cancer, low expression of *BMP5* is associated with poor prognosis in breast, colorectal, lung and bladder cancer. But in ovarian cancer, low expression of *BMP5* is positively correlated with overall survival. Prognostic analysis on the brain and CNS cancer, however, did not show any significant correlation between *BMP5* expression and overall patient survival.

**Figure 4:**
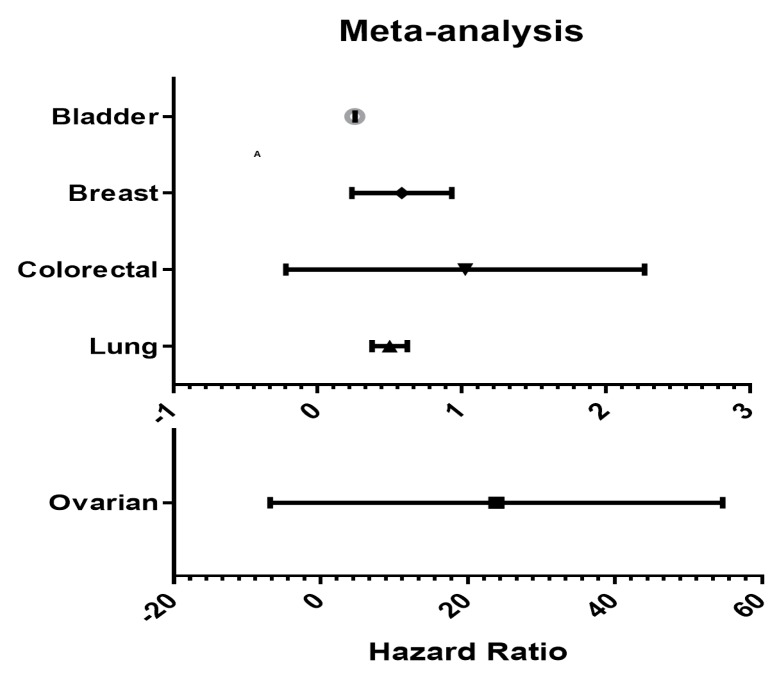
*BMP5* genes in different cancer types (PrognoScan database). The statistically significant hazard ratio for various kinds of cancers was identified from our analyses in Supplementary Table S2 and expressed as the forest plot. The study of survival curve was identified as the threshold of cox *p*-value < 0.05.

In Figure 4, we summarized the prognostic value of *BMP5* expression in various cancers using patient prognosis data from numerous databases with significant Cox *p*-values (*p* < 0.05). Moreover, the Kaplan Meier-plot and PrognoScan showed that low expression of *BMP5* is associated with poor prognosis in bladder, breast, lung, and colorectal cancer compared to the prognosis in ovarian cancer (Figure 3).

### 3.4. Analysis of BMP5 Mutations, Copy Number Alterations and Expression of Mutant mRNA

**Figure 5:**
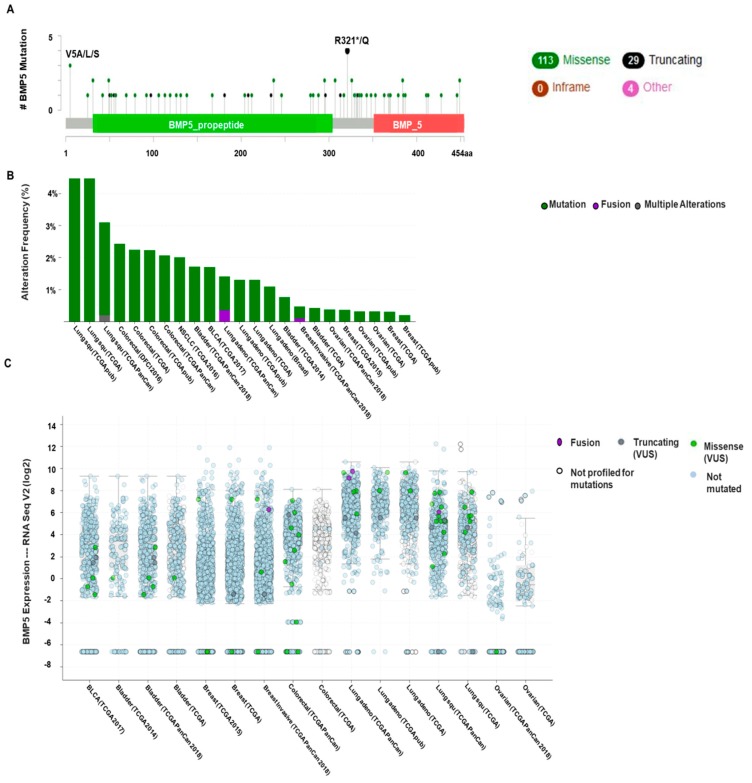
Frequency of mutations, copy number alterations (CNAs) and expression in various types of cancer derived from cBioPortal web. (**A**,**B**), Total 146 mutations were located within amino acids 1 to 454 of *BMP5*. Highest mutations occurred in colorectal cancer, invasive breast cancer at R321*/Q amino acid (total 4 cases) followed by lung squamous carcinoma at V5A/L/S amino acid (total 3 cases). Mutation sites were located in a hotspot in *BMP5*-propeptide and *BMP5* domain. The highest alteration frequency was reported in lung squamous carcinoma datasets. Only datasets containing >100 samples per cancer types are shown. The alteration frequency exhibited mutations (green), fusions (violet) and multiple alterations (grey). (**C**) *BMP5* mRNA expression (RNA Seq V2) in 24 cancer studies generated from the cBioPortal web. Mutations were reported in bladder cancer, breast cancer, colorectal cancer, lung cancer and ovarian cancer. Highest mutated mRNA expression occurred in lung cancer (36 cases). The x-axis is divided according to the cancer types, and Y-axis represents *BMP5* mRNA expression levels. The expression frequency exhibited missense mutations (green), fusions (violet), truncating (deep blue), no mutations (blue), and no profiled mutations (white).

Genetic alterations of *BMP5* in different cancer were studied by utilizing the cBioPortal database. The database queried for the *BMP5* gene mutations in 15,405 number of samples from 26 cancer studies of breast, colorectal, lung, bladder, and ovarian cancer. The gene set or pathway was altered in 2% of the queried samples, with a somatic mutation frequency of 0.9%. In total, 146 mutations, including 70 duplications, were detected in patients with multiple samples (Figure 5A). Mutation sites were located within 1 and 454 amino acids of *BMP5* propeptide and *BMP5* domain. Among these mutations, 113 missense mutations, 29 truncating mutations and four other types of mutations were reported. Highest mutations were reported in breast invasive ductal and colorectal carcinoma and occurred in a hotspot in R321*/Q. Total fourmutations were reported in R321*/Q site, among which missense mutations were found in total 5397 breast invasive ductal carcinoma samples whereas, 837 samples of colorectal adenocarcinoma showed nonsense mutation (Table 1). Total 3 cases of mutation were reported in the hotspot V5A/L/S in lung squamous carcinoma datasets (Figure 5A). Alteration frequency was found highest in lung squamous carcinoma samples (>4%) among five cancer types (Figure 5B). Next, we generated *BMP5* mRNA expression (RNA Seq V2) in 26 cancer studies from the cBioPortal web (Figure 5C). Highest mutated mRNA expression was profiled in lung cancer (36 cases) followed by bladder cancers (14 cases). Mutated *BMP5* proteins were profiled in 11 cases of breast and colorectal cancers, whereas only a single missense mutation was reported in ovarian cancer.

### 3.5. Prediction of Protein–Protein Interaction and Cross-Cancer Analysis of BMP5 Mutations and Copy Number Alterations

**Figure 6:**
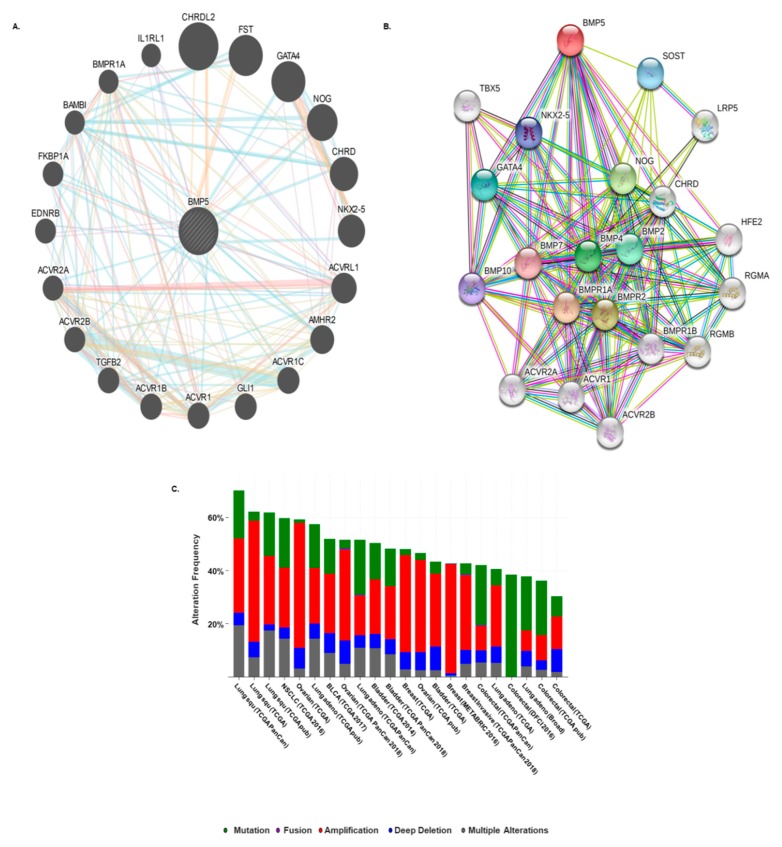
Interaction and co-occurrence functional protein partners of *BMP5*. (**A**,**B**) Predicted structural proteins essential for the functioning of *BMP5* generated from GeneMANIA and STRING web. Circles displayed are indicating nodes. Predicted functional partners are shown after considering co-expression, co-localization, genetic interactions, pathways, physical interactions and predicted shared protein domains. (**C**) The alteration frequency of 31 gene signature from GeneMANIA database (*BMP5*, *HJV*(*HFE2*), *TBX5*, *SOST*, *LRP5*, *RGMA*, *RGMB*, *BMP2*, *BMP4*, *BMP7*, *BMP10*, *FST*, *CHRDL2*, *GLI1*, *BAMBI*, *NOG*, *CHRD*, *NKX2-5*, *IL1RL1*, *GATA4*, *TGFB2*, *EDNRB*, *BMPR1A*, *FKBP1A*, *AMHR2*, *ACVR1*, *ACVRL1*, *ACVR1B*, *ACVR2A*, *ACVR2B*, *ACVR1C*) was determined using cBioPortal web. Datasets containing > 100 samples per cancer type and alteration frequency of >20% are shown. Highest alterations were reported in lung squamous carcinoma datasets (>60%). The alteration frequency included mutations (green), fusions (violet), amplifications (red), deep deletions (deep blue) and multiple alterations (grey).

We found the protein–protein interactions (PPI) involving *BMP5* by using GeneMANIA and STRING web, which compiles data on co-expression, co-localization, genetic interactions, pathways involved, physical interaction predictions and shared protein domains. The predicted protein partners of *BMP5* were: *HJV* (*HFE2*-hemochromatosis type 2 juvenile), *TBX5* (T-box 5), *SOST* (sclerostin), *LRP5* (LDL receptor related protein 5), *RGMA* (repulsive guidance molecule family member a), *RGMB* (repulsive guidance molecule family member b), *BMP2* (bone morphogenetic protein 2), *BMP4* (bone morphogenetic protein 4), *BMP7* (bone morphogenetic protein 7), *BMP10* (bone morphogenetic protein 10), *FST* (follistatin), *CHRDL2* (chordin-like 2), *GLI1* (GLI family zinc finger 1), *BAMBI* (BMP and activin membrane bound inhibitor), *NOG* (noggin), *CHRD* (chordin), *NKX2-5* (NK2 homeobox 5), *IL1RL1* (interleukin 1 receptor like 1), *GATA4* (GATA binding protein 4), *TGFB2* (transforming growth factor beta 2), *EDNRB* (endothelin receptor type B), *BMPR1A* (bone morphogenetic protein receptor type 1A), *FKBP1A* (FK506 binding protein 1A), *AMHR2* (anti-mullerian hormone receptor type 2), *ACVR1* (activin A receptor type 1), *ACVRL1* (activin A receptor like type 1), *ACVR1B* (activin A receptor type 1B), *ACVR2A* (activin A receptor type 2A), *ACVR2B* (activin A receptor type 2B) and *ACVR1C* (activin A receptor type 1C) (Figure 6A,B). Thus, these predicted interacting protein partners of *BMP5* might be involved in the regulation of *BMP5*-mediated cancer progression and prognosis.

We also analyzed the mutations and copy number alterations (CNAs) in the 30 predicted interacting protein partners of *BMP5* by using the ONCOPRINT feature of cBioPortal database. Queried genes were altered in 6751 (38%) of queried patients in 6825 (37%) of queried samples. Highest alteration frequency was found in lung squamous carcinoma (>60%) whereas, colorectal cancer exhibited the lowest alteration (<40%) among all of the query cancer samples (Figure 6C). Among the 30 datasets containing above 100 patient samples, *CHRD* showed the highest alteration frequency (8%) followed by *HEF-2* and *TGFB2* (6%) whereas, *SOST* exhibited the lowest frequency (0.8%) in CNA (Figure S1). The percentage of alteration frequency varied from 0.8–8.0% for individual genes.

### 3.6. Co-occurrence Analysis of Functional Protein Partners of BMP5 in Cancers

We determined the correlation significance of the functional protein partners of *BMP5* by using the co-occurrence analysis sub-tool of cBioPortal, which is based on Fisher′s exact test. In total, 11 genes reported significant co-occurrence of alteration associated with *BMP5* (Table 2). All the genes exhibited *p*-value of < 0.001 and log odds ratio (LOR) of > 1.

### 3.7. Co-expression Heatmap of Functional Protein Partners of BMP5 in Cancers

**Figure 7:**
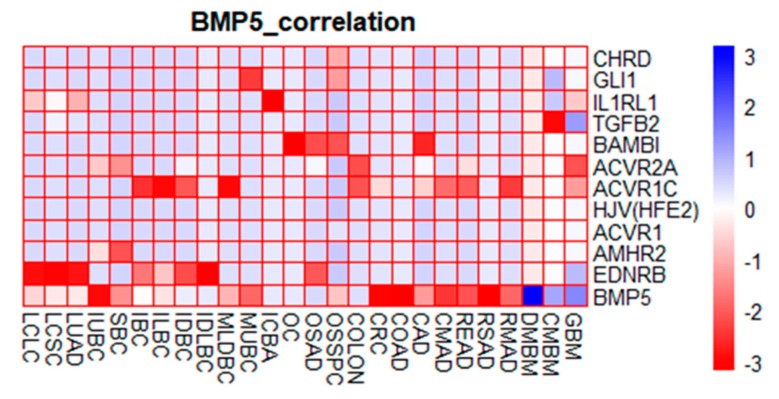
A heatmap visualizing co-expression profile of *BMP5* in breast, colorectal, lung, bladder, ovarian carcinoma and blastoma. *BMP5* is co-expressed with the indicated genes across 3 lung, 7 breast, 8 colorectal, 3 blastoma, 2 bladder, and 1 ovarian cancer sample types. Linear branches on the left and top are clustering tree grouping similar expression pattern of *BMP5* gene in different cancer types. The color scale indicates the expression value (Dark blue indicate higher expression, red indicates lower gene expression). The heat map was generated with custom scripts based on pheatmap function as available in the ‘gplots’ Bioconductor package of R.

We visualized the global features of our gene co-expression data of *BMP5* functional protein partners by conducting hierarchical clustering (Figure 7, Supplementary Table S4).

Hierarchical clustering of expression fold-change values of *BMP5* and 11 functional protein partners in 6 different cancers provides an overview of functional correlation. The analysis showed that *BMP5* was significantly co-expressed with *ACVR1C* in mixed lobular and ductal breast, invasive lobular breast, colon, rectal mucinous, cecum mucinous and rectum adenocarcinoma. *BAMBI*, on the other hand, showed weak co-expression in colon adenocarcinoma and ovarian serous surface papillary carcinoma. *GLI1* showed a significant level of co-expression in mucinous breast carcinoma and ovarian serous surface papillary carcinoma, whereas *AMHR2*, *CHRD*, and *ACVR2A* showed a similar pattern in ovarian serous papillary carcinoma, superficial bladder carcinoma and infiltrating urothelial bladder carcinoma. *IL1RL1* exhibited similar down-regulation in large cell lung carcinoma and lung adenocarcinoma. In contrast, IL1RL1, TGFB2, GLI1, and EDNRB was over-expressed in classical, and desmoplastic medulloblastoma, and glioblastoma.

### 3.8. Differentially Expressed Genes with BMP5 Expression in Five Types of Cancers and Their Function

**Figure 8:**
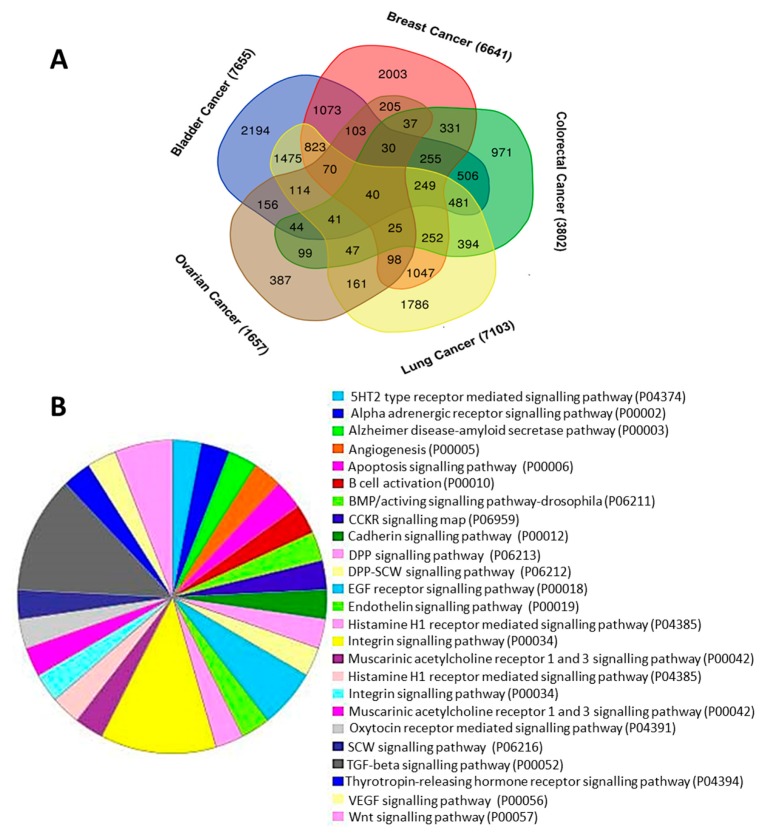
Analysis of genes that positively correlated with *BMP5* and their pathways. (**A**) Analysis of positively correlated genes of *BMP5* using the R2: Genomics Analysis and Visualization Platform. Venn diagram of genes positively correlated to *BMP5*, showing coincident genes in breast, colorectal, lung, bladder, and ovarian cancers. (**B)** Pathway analysis of *BMP5* using PANTHER and subsequent classification based on their pathways; *p* < 0.05.

We aimed to find the potential signalling mechanism involved with *BMP5* expression in cancers. To investigate the *BMP5*-related pathways that might commonly play a role in various cancers, we analyzed transcriptome datasets from five different types of cancers, namely, breast, colon, lung, bladder, and ovarian cancer using TCGA and RSEM datasets through the R2: Genomics Analysis and Visualization Platform. Forty differently expressed genes (DEGs) were commonly up-regulated with *BMP5* in five selected cancers derived from the Venn diagram (Figure 8A).

In our query, a total of 2709 genes were commonly found to be differentially expressed in at least three out of five cancer types. Later we queried for the 11 significantly co-occurrent genes found in previous analysis (Table 2). In our query, *EDNRB* is shown to be co-expressed with *BMP5* in bladder, breast, colorectal and lung cancer. *ACVR2A*, *ACVR1C*, *BMP4*, and *CHRDL2*, on the other hand, was found to be commonly co-expressed with *BMP5* in bladder, breast and lung cancer. *GLI1* also shown co-expression within breast, lung and ovarian cancer datasets. *CHRD*, *NKX2-5* were also co-expressed in bladder and ovarian cancer datasets whereas *BAMBI* was only reported in breast and lung datasets. Next, the common Down-regulated DEGs obtained from R2 and cBioPortal co-occurrence was classified using PANTHER pathway analysis (Figure 8B and Figure S2) and the GOnet: Gene Ontology Analysis tool (Supplementary Table S5). *BMP5* correlated gene cluster affected 33 pathways and was involved in diverse roles such as; TGF-beta signalling pathway, Gonadotropin releasing hormone receptor pathway, EGF-receptor and EGF signalling pathway. We showed that cellular process, cell proliferation and metabolic process are the main biological functions associated with *BMP5* (Figure S2A). In addition, binding and catalytic activities were the main molecular function associated with *BMP5* (Figure S2B). Cell, membrane and protein-containing complex were the major three cellular components associated with *BMP5* gene clusters. Categories obtained from analysis using GOnet: Gene Ontology Analysis tool also contains terms related to signal transduction, gene expression regulation along with cellular proliferation and differentiation, metabolic process regulation, transcriptional and translational regulation and system development (Supplementary Table S5). As conveyed by these results, *BMP5* could be associated with certain key signaling pathways related to cellular proliferation, differentiation, metabolism and post-transcriptional control corresponding to cancer progression.

We want to draw attention to the possibility of eventually being able to derive signaling pathways based on PPI analysis. In our study, the gene list attained by using R2 is characteristically different from the gene list found from PPI investigation. R2 analysis represents the co-expression whereas the latter symbolizes the physical network. On one hand, the correlated genes are pair of genes that shows a similar expression pattern across samples, since the level of transcription of two co-expressed genes rises and fall together across samples. Networks of gene co-expression are of biological interest since co-expressed genes are controlled by the same transcriptional regulatory mechanism, functionally related, or members of the same pathway or protein complex. On the other hand, PPIs are the physical contacts of high specificity established between two or more protein molecules as a result of biochemical events stimulated by electrostatic forces that include hydrophobic effects. On that, we have used the PPI and list of correlated genes for two different purposes. Since genetic alterations are a type of inherent physical manipulation, we used PPI to investigate mutations and CNAs in our query genes. In contrast, we used correlated genes to gain some insights into the signaling pathways.

## 4. Discussion

*BMP5* is a member of TGF-β superfamily and is known to play a regulatory role in the metastasis of lung, thymus, bone marrow, spleen, skeletal muscle, heart, kidney, pancreas, and prostate tissues [53]. It is involved in multivariate disease development and suppression process. It was found in various cases of cancers including breast cancer [20], lung cancer [17], colorectal cancer [12], ovarian cancer [54], bladder cancer [55], skin cancer [56], etc. The mRNA level of *BMP5* was higher in female than the male counterparts in non-small-cell lung cancer (NSCLC) [17]. The overexpressed *BMP5* mRNA has also been found in the squamous cell carcinoma. Conversely, down-regulation was detected in adrenocortical carcinoma cells [9,57]. It plays a role as a tumor suppressor in myeloma, adrenocortical carcinoma, and breast cancer [20,58]. *BMP5* inhibits the proliferation and promote migration and invasion of pancreatic cancer cell and thus exhibit a biphasic role [59].

We transact a multi-omics analysis to understand the potency of *BMP5* as an oncogene or prognostic marker gene in different cancers. To determine the role of *BMP5* in cancer, we have explicitly evaluated the expression pattern of genes in the cancers. normal tissues by using defined parameters through computational analysis. In ONCOMINE and GENT database analysis, we found differential expression pattern depending on cancer cell types. The *BMP5* gene expression in breast, bladder, colorectal, lung and ovarian cancer cells was found lower in compared to normal cells, whereas, it was higher in the brain and CNS cancer. Then we performed distinct analysis for revealing the role of *BMP5* in breast, bladder, colorectal, lung, and ovarian cancers. In the case of breast cancer, *BMP5* expression was low using ONCOMINE, UALCAN, and GEPIA database. Our further expression analysis using these analyzing tools exhibited similar results for bladder, colorectal, lung, and ovarian cancers. This results also support the previous results where Deng et al. reported lower *BMP5* expression in NSCLC tissues by quantitative real-time PCR analysis [17].

Overall, our analysis has shown that *BMP5* was significantly down-regulated in several cancer datasets. However, we were unable to identify a consistent clinical outcome pattern relating to the clusters. It is important to note that the survival data of several TCGA data set are limited by the length of follow up [60,61].

To understand the role of *BMP5* in cancer more definitely, we investigated the prognostic status of *BMP5* in breast, bladder, colorectal, lung, and ovarian cancer. We utilized the PrognoScan database to find out the association of *BMP5* mRNA expression and overall survival rate. The inquisition reports that lower *BMP5* mRNA expression was associated with lower overall survival rate in breast cancer and lung cancer. In breast cancer, Blimp-1 plays an inevitable role in TGF-β1 mediated epithelial-to-mesenchymal transition (EMT) through the suppression of *BMP5* expression [20]. In colorectal cancer due to the conflicting results, the role of *BMP5* in colorectal cancer was not clear. According to the study of William et al. *BMP5* inuced ERBB4 is over-expressed in human CRC, and in experimental systems enhances the survival and growth of cells driven by Ras and/or WNT signaling. Chronic ERBB4 over-expression in the context of, for example, inflammation may contribute to the development of colorectal cancer [62]. Results exhibited an association of lower expression with lower survival rate from four survival curve analysis (Figure 3C,E–G), whereas another one exhibited the opposite result (Figure 3E). Chen et al. observed the down-regulation of *BMP5* in colorectal cancer due to the miR-32 deregulation [14]. The mean results of the analysis of the current investigation suggest the role of *BMP5* as tumor suppressor gene in colorectal cancer. This result also supports the previous findings, where *BMP5* acts as a tumor suppressor in colorectal cancer [12]. Our analysis on ovarian cancer represents the correlation between lower *BMP5* mRNA expression and higher overall survival rate. *BMP2*, *5* and rhBMP9 has been reported to be involved in RAS-MAPK mediated pathway that activates p65 cytokine and during tumorigenesis and thus proliferate ovarian cancer [63,64]. On the other hand, investigation on bladder cancer prognosis relevance using PROGgene V2 database recommends a positive relation between lower *BMP5* expression and lower relapse-free survival. This consistent trend in *BMP5* expression in cancers suggests a commonality among different cancer types, where *BMP5*-related molecular pathways may be functional.

Cancer progression is a series of histopathological process, which is influenced by somatically acquired genetic, epigenetic, transcriptomic and proteomic alterations [65,66,67]. Somatic loss-of-function or gain-of-function alterations ensue in particular genomic regions which engaged in potential inhibitory or carcinogenic effects, respectively [68,69]. Therefore, we exploited the cBioPortal web tool to exhibit the Copy Number Alterations, mutations and mutant mRNA expressions. Missense and truncating mutations pre-eminently occurred within protein-coding sequences [13]. Ho et al. reported a novel missense mutation in *BMP5* transcript with a G-to-A substitution in RXXR cleavage consensus site. The G-to-A mutation at nucleotide 932 cleaves the nucleotides encoding arginine (CGA) residue and thus destroys the Taq I polymerase restriction site resulting in the blockage of the post-translational processing of the *BMP5* protein [70]. In our analysis, we found diverse missense and truncating mutations in the *BMP5* protein-coding sequence while exposed to cancer cells. This study revealed mutations in breast invasive ductal and colorectal carcinoma in a hotspot at position R321*/Q and position V5A/L/S in lung squamous carcinoma between *BMP5*-propeptide and *BMP5* domain. Similar results of mutation at R321*/Q stop-codon (4 cases) was also found in a previous study which may impact cleavage of the protein to the mature secreted form [13]. Highest alteration frequency was obtained from lung squamous carcinoma. Another *BMP5* mRNA expression (RNA Seq V2) analysis via cBioPortal web found a higher mutation rate in lung cancer and then in bladder cancers. These mutations may have played an essential role in cancer progression and prognosis.

It has been reported that PPI is associated with various biological processes, such as signal transduction and disease development [71]. To predict their functional protein partner, we exploited GeneMANIA and STRING web server. These interacted proteins have a role in the regulation of tumor progression. A total of 30 proteins were determined as protein partners, and these interacting proteins were used to detect copy number alterations and mutations through the cBioPortal tool. From the lowest to the highest dominance hierarchy, the ratio of alteration ranged over >25% to >60%. The copy number alteration frequency of each gene showed that *CHRD* obtains the highest alteration frequency with a percentage of 8% whereas *SOST* occupied the lowest position. After that, the correlation between each gene and *BMP5* was evaluated significantly via cBioPortal under specific parameter. This analysis reports the co-occurrence genes association with *BMP5* during tumorigenesis. To determine the hierarchy, we also observed a heatmap characterized by fold-change values of the 11 functional partners of *BMP5*. GLI1 and ACVR1C were found to be down-regulated in breast, colorectal, and ovarian cancer. Previous reports also suggest that both GLI1 and ACVR1C are down-regulated in breast cancer [72]. ACVR1C down-regulation in colorectal cancer was reported in several studies [73,74]. However, previous studies reported that GLI1 overexpression in malignant ovarian epithelial tumor enables tumor metastasis and increase risk in ovarian cancer patient [75,76]. These reports are not similar to our findings in Figure 7 and Table 1 of S2 File. Previous studies found that BAMBI regulates CRC and NSCLC metastasis by connecting the Wnt/beta-catenin and TGF-β-signalling pathways which validate our findings [77,78]. BAMBI is also reported to be overexpressed in breast and ovarian cancer and co-translocate with Smads into the nucleus upon TGF-β treatment [79,80]. Circular ACVR2A expression was found to be suppressed in bladder cancer tissues and cell lines and has a functional role in bladder cancer inhibition and metastasis [81]. CHRD was found to be significantly under-expressed in ovarian cancer compared to normal tissues suggesting its potential of regulating BMP activity in normal ovarian surface epithelium physiology [82]. Several reports on IL1RL1 down-regulation in lung cancer also validates our findings in this study [83,84]. Here, our findings validate the co-expression significance of *BMP5* with its predicted functional protein partners in several cancers.

Finally, we determined the genes correlated with *BMP5* in five types of cancer (breast, lung, colorectal, bladder, and ovarian) in which this TGF-β family member is significantly down-regulated by using R2 platform. For *BMP5*, a large number of positively-correlated genes were found in breast, colon, lung, bladder, and ovarian cancer. Of those genes, 40 were common in all cancers along with 2709 genes common in at least three out of five cancers. R2 analysis also validates our findings from cBioPortal analysis and heatmap of fold change values of the 11 protein partners. In addition, we used the PANTHER pathway and GOnet: Gene Ontology Analysis tool to determine the GO and pathways associated with these commonly correlated genes for *BMP5*. According to the pathway analysis, ACVR1C, ACVR2A, AMHR2, TGFB2, BAMBI, and *BMP5* were predominantly found to be involved in TGF- β signalling and gonadotrophin releasing hormone receptor pathways. The co-expression analysis shows that *BMP5* expression is positively correlated with transmembrane receptor protein serine/threonine kinase pathway regulation, cellular proliferation and differentiation, metabolic process regulation, transcriptional and translational regulation, regulation of cell death, and system development (Supplementary Table S5). GO analysis completely validates our findings on co-expression and pathway regulation of functional protein partners of *BMP5*. However, their underlying mechanism in development of cancers and the effects on clinical outcomes remains unknown.

## 5. Conclusions

In this mining study, we used several online bioinformatics platform and web tools to systematically analyze the expression, mutations and CNAs, correlated genes, and prognostic value of *BMP5* in human various cancers. The multi-omics analysis revealed that *BMP5* is down-regulated distinctively and is negatively correlated with clinical outcomes in breast colorectal, lung, and bladder, except ovarian cancer. Ovarian cancer is positively correlated. The present findings also reveal the importance of *BMP5* expression and possible *BMP5*-related pathways in human various cancers progression. Overall, our analysis may provide valuable insights into *BMP5* as a novel biomarker and a potential therapeutic target for various human cancers and thus, will assist in transforming genomic knowledge into clinical practice. Hence, the specific roles, detailed molecular mechanisms, and clinical significance of *BMP5* in cancer progression and prognosis deserve further investigation

## Figures and Tables

**Table 1:** Data analysis of the *BMP5* gene in three different cancer types derived from cBioPortal for Cancer Genomics.

Cancer Dataset	Cancer Type	Protein Change	Mutation Type	Number of Mutation in Sample	Sample ID
BRIC (TCGA, PanCancer Atlas)	Breast invasive ductal carcinoma	*R321Q*	Missense	5397	TCGA-AN-A046-01
COAD (DFIC, Cell Reports 2016)	Colorectal adenocarcinoma	R321*	Nonsense	180	coad_read_dfci_2016_1250
COAD (DFIC, Cell Reports 2016)	Colorectal adenocarcinoma	R321*	Nonsense	513	coad_read_dfci_2016_2946
COAD (TCGA, PanCancer Atlas)	Colon adenocarcinoma	R321*	Nonsense	144	TCGA-D5-6533-01

Total of five mutations reported in amino acid R321 in breast cancer and colorectal cancer datasets. Total 10,507 mutations were reported in five samples.

**Table 2:** Mutual exclusivity analysis reporting co-occurrence of alterations in the *BMP5* gene signature was investigated using cBioPortal for Cancer Genomics.

Co-occurrence Gene	*p*-Value	Log Odds Ratio	Tendency	Significance
*CHRD*	<0.001	2.139	Co-occurrence	Significant
*GLI1*	<0.001	2.153	Co-occurrence	Significant
*IL1RL1*	<0.001	2.392	Co-occurrence	Significant
*TGFB2*	<0.001	2.136	Co-occurrence	Significant
*BAMBI*	<0.001	2.138	Co-occurrence	Significant
*ACVR2A*	<0.001	2.046	Co-occurrence	Significant
*ACVR1C*	<0.001	2.350	Co-occurrence	Significant
*HJV*(*HFE2*)	<0.001	1.866	Co-occurrence	Significant
*ACVR1*	<0.001	2.250	Co-occurrence	Significant
*AMHR2*	<0.001	2.208	Co-occurrence	Significant
*EDNRB*	<0.001	1.504	Co-occurrence	Significant

Total 30 cancer datasets containing samples for five cancer types were investigated. Significant co-occurrence was reported only for the pairs resulting with *p* < 0.001.

## Supplementary Materials

The following are available online at https://www.mdpi.com/2227-9059/8/2/19/s1, Table S1: Dataset query results of *BMP5* expression in cancer and normal tissue samples derived from GENT database. Table S2: The significant changes of *BMP5* expression in normal tissues and cancer tissue were derived from the ONCOMINE database. Table S3: The relationship between *BMP5* expression and survival in various cancer patients. Table S4: Fold change values of functional protein partners of *BMP5* (Oncomine). Table S5: Gene ontology (GO) terms obtained from genes positively correlated with *BMP5* (GOnet). Table S6: List of correlated genes of *BMP5* in five cancer types obtained from R2: Genomics Analysis and Visualization Platform. Table S7: Analysis of positively correlated genes of *BMP5* using the R2: Genomics Analysis and Visualization Platform. Figure S1: Copy number alteration frequency of each gene of the *BMP5* cluster from GeneMANIA and STRING database. A total of 17815 patients / 18300 samples in 30 studies were queried. Queried genes were altered in 6751 (38%) of queried patients in 6825 (37%) of queried samples. The alterations include inframe mutations-unknown significance (brown), missense mutations-putative driver (deep green), missense mutations-unknown significance (green), truncating mutations-putative driver (black), truncating mutations-unknown significance (grey), fusions (violet), amplifications (red), deep deletions (deep blue), no alterations (light grey) and not profiled cases (white). Figure S2: Pathway analysis of *BMP5* using PANTHER pathway tool. A. A total of 49 biological processes were reported and distributed in eight categories. B. A total of 26 cellular components were reported and distributed in four categories. C. A total of 34 molecular functions were reported and distributed in seven categories. * *p* < 0.05.

## Abbreviations

ADC	Adenocarcinoma
C	Carcinoma
CAD	Colon adenoma
CCC	Clear cell carcinoma.
CEAD	Cecum adenocarcinoma
CMAD	Cecum mucinous adenocarcinoma
CMAD	Colon mucinous adenocarcinoma
CMBM	Classical medulloblastoma
CNS	Central nervous system
Colon	Colon carcinoma
COAD	Colon adenocarcinoma
CRA	Colorectal adenomacarcinoma
CRAD	Colorectal adenocarcinoma
CRC	Colorectal carcinoma
DMBM	Desmoplastic medulloblastoma
EMC	Endometroid carcinoma
GBM	Glioblastoma
IBC	Invasive breast carcinoma
IDBC	Invasive ductal breast carcinoma
ILBC	Invasive lobular and ductal breast carcinoma
LCLC	Large cell lung carcinoma
LUAD	Lung adenocarcinoma
LUSC	Lung squamous cell carcinoma
MC	Mucinous carcinoma
MixC	Mixed carcinoma
MLDBC	Mixed lobular and ductal breast carcinoma
MPC	Micropapillary carcinoma
MUBC	Mucinous breast carcinoma
OC	Ovarian cancer
OSAD	Ovarian surface adenocarcinoma
OSSPC	Ovarian serous surface papillary carcinoma
RAD	Rectal adenocarcinoma
READ	Rectum mucinous adenocarcinoma
RMAD	Rectal mucinous adenocarcinoma
RSAD	Rectosigmoid adenocarcinoma
SBC	Serous breast carcinoma
TCGA	The cancer genome atlas
UC	Undifferentiated carcinoma
