# Supplementary material for: A Multi-Omics Analysis of Bone Morphogenetic Protein 5 (BMP5) mRNA Expression and Clinical Prognostic Outcomes in Different Cancers Using Bioinformatics Approaches
Source: Biomedicines. 2020 Jan 21;8(2):19. doi: 10.3390/biomedicines8020019 (PMC7168281; doi:10.3390/biomedicines8020019)
Supplement: Supplementary file 1 [file biomedicines-08-00019-s001.zip › biomedicines-664747-supplementary new11/Supplementary files/supplementary materials.docx]

**Supplementary Materials: A Multi−Omics Analysis of Bone Morphogenetic Protein 5 (*BMP5) mRNA* Expression and Clinical Prognostic Outcomes in Different Cancers Using Bioinformatics Approaches**

**Supplementary Table S2.** The significant changes of BMP5 expression in normal tissues and cancer tissue was derived from the Oncomine database.

| Cancer Types | Dataset | Normal (Cases) | Cancer (Cases) | Fold Change | t−Test | *p*−Value | Rank (%) |
| --- | --- | --- | --- | --- | --- | --- | --- |
| Breast cancer | TCGA | Breast (61) | Mucinous breast carcinoma (4) | −4.712 | −16.423 | 1.03 × 10^−11^ | 1 |
|  |  |  | Mixed lobular and ductal breast (7) | −3.291 | −8.966 | 6.13 × 10^−7^ | 2 |
|  |  |  | Invasive lobular breast (36) | −2.452 | −7.300 | 1.73 × 10^−10^ | 6 |
|  |  |  | Invasive ductal breast (389) | −2.705 | −12.060 | 1.54 × 10^−21^ | 9 |
|  |  |  | Invasive breast (76) | −2.624 | −7.333 | 1.25 × 10^−11^ | 10 |
| Lung cancer | Hou Lung | Lung (65) | Squamous cell lung carcinoma (27) | −7.108 | −17.953 | 2.72 × 10^−25^ | 1 |
|  |  |  | Large cell lung carcinoma (19) | −5.177 | −9.135 | 1.51 × 10^−9^ | 5 |
|  |  |  | Lung adenocarcinoma (45) | −3.267 | −8.133 | 7.37 × 10^−12^ | 6 |
|  | Beer | Lung (10) | Lung adenocarcinoma (86) | −6.923 | −6.680 | 5.58 × 10^−8^ | 5 |
|  | Okayama | Lung (20) | Lung adenocarcinoma (226) | −2.717 | −9.058 | 2.50 × 10^−12^ | 4 |
|  | su | Lung (30) | Lung adenocarcinoma (27) | −2.898 | −5.034 | 5.07 × 10^−6^ | 7 |
| Colorectal cancer | Hong | Colon (12) | Colorectal carcinoma (70) | −11.931 | −14.738 | 1.12 × 10^−21^ | 1 |
|  | Sabates−Bellver | Ascending colon (4) | Rectal adenoma (7) | −2.150 | −6.735 | 1.38 × 10^−5^ | 2 |
|  |  | Descending colon (5) | Colon adenoma (25) | −4.671 | −8.962 | 1.47 × 10^−10^ | 2 |
|  |  | Rectum (7) |  |  |  |  |  |
|  |  | Sigmoid colon (15) |  |  |  |  |  |
|  |  | Transverse colon (1) |  |  |  |  |  |
|  | TCGA | Colon (19) | Colon mucinous adenocarcinoma (22) | −5.767 | −11.106 | 6.54 × 10^−13^ | 3 |
|  |  | Rectum (3) | Rectal mucinous adenocarcinoma (6) | −3.081 | −8.600 | 5.01 × 10^−7^ | 3 |
|  |  |  | Colon adenocarcinoma (101) | −3.654 | −13.661 | 1.63 × 10^−18^ | 4 |
|  |  |  | Rectal adenocarcinoma (60) | −3.248 | −11.305 | 1.18 × 10^−16^ | 5 |
|  |  |  | Cecum adenocarcinoma(22) | −3.437 | −7.453 | 5.96 × 10^−9^ | 10 |
|  | Skrzypczak | Colorectal tissue (24) | Colorectal carcinoma (36) | −2.393 | −7.911 | 1.06 × 10^−10^ | 3 |
|  | Gaedcke | Rectum (65) | Rectal adenocarcinoma (65) | −2.639 | −13.117 | 3.17 × 10^−24^ | 4 |
|  | Kaiser | Colon (5) | Colon mucinous adenocarcinoma (13) | −2.259 | −8.935 | 1.53 × 10^−5^ | 5 |
|  |  |  | Rectosigmoid adenocarcinoma (10) | −2.007 | −8.102 | 9.17 × 10^−5^ | 6 |
|  | Skrzypczak 2 | Colon (10) | Colon carcinoma (5) | −8.655 | −7.272 | 3.22 × 10^−6^ | 6 |
|  |  |  | Colon adenoma (5) | −4.635 | −6.104 | 4.71 × 10^−5^ | 8 |
| Bladder cancer | Lee | Bladder mucosa(68) | Infiltrating bladder urothelial carcinoma (62) | −2.140 | −7.412 | 8.99 × 10^−12^ | 1 |
|  |  |  | Superficial bladder cancer (126) | −2.167 | −8.775 | 5.48 × 10^−14^ | 2 |
|  | Dyrskjot 3 | Bladder (9) | Infiltrating bladder urothelial carcinoma (13) | −2.108 | −6.972 | 2.57 × 10^−7^ | 2 |
|  |  | Bladder mucosa (5) |  |  |  |  |  |
|  | Sanchez−Carbayo Bladder 2 | Bladder (48) | Infiltrating bladder urothelial carcinoma (81) | −4.669 | −7.034 | 1.99 × 10^−10^ | 7 |
| Ovarian cancer | Welsh | Ovary (4) | Ovarian serous surface papillary carcinoma (28) | −8.241 | −5.133 | 1.05 × 10^−5^ | 5 |
| Brain and CNS cancer | Pomeroy | Cerebellum (4) | Desmoplastic Medulloblastoma (14) | 79.550 | 8.043 | 5.88 × 10^−7^ | 22 |
|  |  | Cerebellum (4) | Classic Medulloblastoma (46) | 6.830 | 5.467 | 9.11 × 10^−7^ | 228 |
|  |  | Brain (4) | Glioblastoma (27) | 4.631 | 10.859 | 5.87 × 10^−6^ | 729 |

**Supplementary Table S3.** The relationship between BMP5 expression and the survival in various cancer patients.

| Cancer Type | Dataset | Endpoint | Array Type | Probe ID | N | Cox *p*−Value | Hr [95% ci−Low ci−upp] |
| --- | --- | --- | --- | --- | --- | --- | --- |
| Breast cancer | GSE19615 | Distant metastasis free survival | HG−U133_Plus_2 | 205430_at | 115 | 0.019437 | 0.34 [0.14–0.84] |
|  | GSE12276 | Relapse free survival | HG−U133_Plus_2 | [205431_s_at](http://dna00.bio.kyutech.ac.jp/PrognoScan-cgi/PrognoScan.cgi?TEST_NUM=89&DATA_POSTPROCESSING=None&MODE=SHOW_GRAPH&TITLE=Prognostic+value%20of%20BMP5%20mRNA%20expression%20in%20Breast%20cancer&PROBE_ID=4014879#_blank) | 204 | 0.027596 | 0.83 [0.70–0.98] |
| Colorectal cancer | GSE17536 | Disease specific survival | HG−U133_Plus_2 | 205431_s_at | 177 | 0.006737 | 0.26 [0.10–0.69] |
|  | GSE17537 | Overall survival | HG−U133_Plus_2 | 205430_at | 55 | 0.010919 | 3.22 [1.31–7.90] |
|  | GSE17536 | Overall survival | HG−U133_Plus_2 | 205431_s_at | 177 | 0.012800 | 0.36 [0.16–0.80] |
|  | GSE14333 | Disease free survival | HG−U133_Plus_2 | [205430_at](http://dna00.bio.kyutech.ac.jp/PrognoScan-cgi/PrognoScan.cgi?TITLE=Prognostic+value%20of%20BMP5%20mRNA%20expression%20in%20Colorectal%20cancer&MODE=SHOW_GRAPH&DATA_POSTPROCESSING=None&TEST_NUM=68&PROBE_ID=4014878#_blank) | 226 | 0.032568 | 0.81 [0.66–0.98] |
|  | GSE17536 | Disease specific survival | HG−U133_Plus_2 | 205430_at | 177 | 0.047128 | 0.48 [0.23–0.99] |
| Lung cancer | [GSE31210](http://dna00.bio.kyutech.ac.jp/PrognoScan-cgi/PrognoScan.cgi?PROBE_ID=4014878&TITLE=Prognostic+value%20of%20BMP5%20mRNA%20expression%20in%20Lung%20cancer&MODE=SHOW_GRAPH&TEST_NUM=108&DATA_POSTPROCESSING=None#_blank) | Relapse free survival | HG−U133_Plus_2 | [205430_at](http://dna00.bio.kyutech.ac.jp/PrognoScan-cgi/PrognoScan.cgi?PROBE_ID=4014878&TITLE=Prognostic+value%20of%20BMP5%20mRNA%20expression%20in%20Lung%20cancer&MODE=SHOW_GRAPH&TEST_NUM=108&DATA_POSTPROCESSING=None#_blank) | 204 | 0.000007 | 0.59 [0.47–0.74] |
|  | GSE31210 | Overall survival | HG−U133_Plus_2 | 205430_at | 204 | 0.000138 | 0.55 [0.41–0.75] |
|  | Jacob−00182−MSK | Overall survival | HG−U133A | 205431_s_at | 104 | 0.001581 | 0.29 [0.13–0.62] |
|  | jacob−00182−MSK | Overall survival | HG−U133A | 205430_at | 104 | 0.004857 | 0.57 [0.38–0.84] |
|  | GSE31210 | Overall survival | HG−U133_Plus_2 | 205431_s_at | 204 | 0.044497 | 0.51 [0.27–0.98] |
| Ovarian cancer | DUK × 10−OC | Overall survival | HG−U133A | 205431_s_at | 133 | 0.011589 | 45.62 [2.35–885.66] |
|  | DUK × 10−OC | Overall survival | HG−U133A | 205430_at | 133 | 0.034529 | 2.11 [1.06–4.21] |
| Brain and CNS cancer | GSE7696 | Overall survival | HG−U133_Plus_2 | 205431_s_at | 70 | 0.704812 | 1.11 [0.64–1.92] |
|  | GSE7696 | Overall survival | HG−U133_Plus_2 | 205430_at | 70 | 0.969217 | 1.01 [0.70–1.46] |

**Supplementary Table S5:** Gene ontology (GO) terms obtained from genes positively correlated with *BMP5* (GOnet)

| GO_term_ID | GO_term_def | P | P_FDR_adj | NofGenes | Genes |
| --- | --- | --- | --- | --- | --- |
| GO:0007178 | transmembrane receptor protein serine/threonine kinase signaling pathway | 8.00 × 10^−10^ | 8.71 × 10^−6^ | 9 | ACVR1\|ACVR1C\|ACVR2A\|AMHR2\|ATOH8\|BAMBI\|BMP5\|CHRD\|TGFB2 |
| GO:0007167 | enzyme linked receptor protein signaling pathway | 6.38 × 10^−08^ | 3.52 × 10^−4^ | 12 | ACVR1\|ACVR1C\|ACVR2A\|AMHR2\|ATOH8\|BAMBI\|BMP5\|BTC\|CHRD\|EPHA7\|KIT\|TGFB2 |
| GO:0048598 | embryonic morphogenesis | 6.07 × 10^−07^ | 1.67 × 10^−3^ | 10 | ACVR1\|ACVR2A\|ATOH8\|BMP5\|CHRD\|FREM2\|GLI1\|OSR1\|SLC44A4\|TGFB2 |
| GO:0030509 | BMP signaling pathway | 2.18 × 10^−6^ | 3.43 × 10^−3^ | 5 | ACVR1\|ACVR2A\|AMHR2\|BMP5\|CHRD |
| GO:0007166 | cell surface receptor signaling pathway | 3.85 × 10^−06^ | 4.45 × 10^−03^ | 18 | ACVR1\|ACVR1C\|ACVR2A\|AMHR2\|ATOH8\|BAMBI\|BMP5\|BTC\|CHRD\|EDNRB\|EPHA7\|GLI1\|IL1RL1\|KCNA5\|KIT\|PRKCD\|SOD2\|TGFB2 |
| GO:0007154 | cell communication | 3.87 × 10^−06^ | 4.45 × 10^−03^ | 28 | ACVR1\|ACVR1C\|ACVR2A\|AMHR2\|ARHGAP28\|ATOH8\|BAMBI\|BMP5\|BTC\|CHRD\|CLEC5A\|EDNRB\|EPHA7\|FREM2\|GLI1\|IL1RL1\|KCNA5\|KIT\|OR2M5\|OR4C12\|OR5F1\|PDE1C\|PDE7B\|PRKCD\|S100A14\|SCN3A\|SOD2\|TGFB2 |
| GO:0071772 | response to BMP | 4.70 × 10^−06^ | 4.45 × 10^−03^ | 5 | ACVR1\|ACVR2A\|AMHR2\|BMP5\|CHRD |
| GO:0071773 | cellular response to BMP stimulus | 4.70 × 10^−06^ | 4.45 × 10^−03^ | 5 | ACVR1\|ACVR2A\|AMHR2\|BMP5\|CHRD |
| GO:0010862 | positive regulation of pathway−restricted SMAD protein phosphorylation | 5.05 × 10^−06^ | 4.45 × 10^−03^ | 4 | ACVR1\|ACVR2A\|BMP5\|TGFB2 |
| GO:0072132 | mesenchyme morphogenesis | 5.95 × 10^−06^ | 4.45 × 10^−03^ | 4 | ACVR1\|BMP5\|OSR1\|TGFB2 |
| GO:0032879 | regulation of localization | 6.05 × 10^−06^ | 4.45 × 10^−03^ | 19 | ACVR1\|ACVR1C\|ATOH8\|BMP5\|BTC\|CHRD\|CLEC5A\|EDNRB\|IL1RL1\|ILDR1\|KCNA5\|KIT\|NECAB2\|OSR1\|PRKCD\|S100A14\|SCN3A\|SOD2\|TGFB2 |
| GO:0007389 | pattern specification process | 7.12 × 10^−06^ | 4.91 × 10^−03^ | 8 | ACVR1\|ACVR1C\|ACVR2A\|AMHR2\|BMP5\|CHRD\|GLI1\|OSR1 |
| GO:0023052 | signaling | 9.94 × 10^−06^ | 6.39 × 10^−03^ | 27 | ACVR1\|ACVR1C\|ACVR2A\|AMHR2\|ARHGAP28\|ATOH8\|BAMBI\|BMP5\|BTC\|CHRD\|CLEC5A\|EDNRB\|EPHA7\|GLI1\|IL1RL1\|KCNA5\|KIT\|OR2M5\|OR4C12\|OR5F1\|PDE1C\|PDE7B\|PRKCD\|S100A14\|SCN3A\|SOD2\|TGFB2 |
| GO:0007165 | signal transduction | 1.04 × 10^−05^ | 6.39 × 10^−03^ | 26 | ACVR1\|ACVR1C\|ACVR2A\|AMHR2\|ARHGAP28\|ATOH8\|BAMBI\|BMP5\|BTC\|CHRD\|CLEC5A\|EDNRB\|EPHA7\|GLI1\|IL1RL1\|KCNA5\|KIT\|OR2M5\|OR4C12\|OR5F1\|PDE1C\|PDE7B\|PRKCD\|S100A14\|SOD2\|TGFB2 |
| GO:0009790 | embryo development | 1.21 × 10^−05^ | 6.97 × 10^−03^ | 11 | ACVR1\|ACVR2A\|ATOH8\|BMP5\|CHRD\|FREM2\|GLI1\|KIT\|OSR1\|SLC44A4\|TGFB2 |
| GO:0060393 | regulation of pathway−restricted SMAD protein phosphorylation | 1.32 × 10^−05^ | 6.97 × 10^−03^ | 4 | ACVR1\|ACVR2A\|BMP5\|TGFB2 |
| GO:0032501 | multicellular organismal process | 1.33 × 10^−05^ | 6.97 × 10^−03^ | 31 | ACVR1\|ACVR1C\|ACVR2A\|AMHR2\|ATOH8\|B3GNT5\|BMP5\|BTC\|CHRD\|CLEC5A\|COL10A1\|COL21A1\|EDNRB\|EPHA7\|FREM2\|GLI1\|KCNA5\|KIT\|KRTAP12−4\|MARVELD2\|OLR1\|OR2M5\|OR4C12\|OR5F1\|OSR1\|PCDH18\|PRKCD\|SCN3A\|SLC44A4\|SOD2\|TGFB2 |
| GO:0090092 | regulation of transmembrane receptor protein serine/threonine kinase signaling pathway | 1.71 × 10^−05^ | 8.58 × 10^−03^ | 6 | ACVR1\|ACVR2A\|BAMBI\|BMP5\|CHRD\|TGFB2 |
| GO:0001501 | skeletal system development | 1.81 × 10^−05^ | 8.65 × 10^−03^ | 8 | ACVR2A\|BMP5\|CHRD\|COL10A1\|COL21A1\|KIT\|OSR1\|TGFB2 |
| GO:0071363 | cellular response to growth factor stimulus | 1.89 × 10^−05^ | 8.66 × 10^−03^ | 8 | ACVR1\|ACVR1C\|ACVR2A\|AMHR2\|BAMBI\|BMP5\|CHRD\|TGFB2 |
| GO:0035295 | tube development | 2.09 × 10^−05^ | 9.22 × 10−^03^ | 10 | ACVR1\|ATOH8\|BMP5\|CHRD\|EDNRB\|EPHA7\|GLI1\|KIT\|OSR1\|TGFB2 |
| GO:0070848 | response to growth factor | 2.88 × 10^−05^ | 1.16 × 10^−02^ | 8 | ACVR1\|ACVR1C\|ACVR2A\|AMHR2\|BAMBI\|BMP5\|CHRD\|TGFB2 |
| GO:0060037 | pharyngeal system development | 3.15 × 10^−05^ | 1.16 × 10^−02^ | 3 | ACVR1\|BMP5\|TGFB2 |
| GO:0046661 | male sex differentiation | 3.26 × 10^−05^ | 1.16 × 10^−02^ | 5 | ACVR2A\|AMHR2\|BMP5\|KIT\|TGFB2 |
| GO:0009888 | tissue development | 3.32 × 10^−05^ | 1.16 × 10^−02^ | 14 | ACVR1\|ACVR2A\|BMP5\|CHRD\|COL21A1\|EDNRB\|EPHA7\|FREM2\|GLI1\|KRTAP12−4\|MARVELD2\|OSR1\|SLC44A4\|TGFB2 |
| GO:0007548 | sex differentiation | 3.48 × 10^−05^ | 1.16 × 10^−02^ | 6 | ACVR2A\|AMHR2\|BMP5\|KIT\|OSR1\|TGFB2 |
| GO:0060429 | epithelium development | 3.78 × 10^−05^ | 1.18 × 10^−02^ | 11 | ACVR1\|BMP5\|CHRD\|EPHA7\|FREM2\|GLI1\|KRTAP12−4\|MARVELD2\|OSR1\|SLC44A4\|TGFB2 |
| GO:2000145 | regulation of cell motility | 3.86 × 10^−05^ | 1.18 × 10^−02^ | 10 | ACVR1\|ACVR1C\|ATOH8\|BMP5\|BTC\|CHRD\|KIT\|S100A14\|SOD2\|TGFB2 |
| GO:0008284 | positive regulation of cell population proliferation | 3.97 × 10^−05^ | 1.18 × 10^−02^ | 10 | BAMBI\|BMP5\|BTC\|CHRD\|EDNRB\|GLI1\|KCNA5\|KIT\|OSR1\|TGFB2 |
| GO:0048729 | tissue morphogenesis | 4.39 × 10^−05^ | 1.24 × 10^−02^ | 8 | ACVR1\|BMP5\|CHRD\|COL21A1\|EPHA7\|FREM2\|OSR1\|TGFB2 |
| GO:0045595 | regulation of cell differentiation | 4.44 × 10^−05^ | 1.24 × 10^−02^ | 14 | ACVR1\|ACVR2A\|ATOH8\|BAMBI\|BMP5\|BTC\|CHRD\|EDNRB\|EPHA7\|GLI1\|KIT\|OSR1\|SOD2\|TGFB2 |
| GO:0010717 | regulation of epithelial to mesenchymal transition | 4.49 × 10^−05^ | 1.24 × 10^−02^ | 4 | ACVR1\|BAMBI\|BMP5\|TGFB2 |
| GO:0048608 | reproductive structure development | 5.27 × 10^−05^ | 1.32 × 10^−02^ | 7 | ACVR2A\|AMHR2\|BMP5\|GLI1\|KIT\|OSR1\|TGFB2 |
| GO:0061458 | reproductive system development | 5.52 × 10^−05^ | 1.32 × 10^−02^ | 7 | ACVR2A\|AMHR2\|BMP5\|GLI1\|KIT\|OSR1\|TGFB2 |
| GO:0042127 | regulation of cell population proliferation | 5.75 × 10^−05^ | 1.35 × 10^−02^ | 13 | ACVR1C\|ATOH8\|BAMBI\|BMP5\|BTC\|CHRD\|EDNRB\|GLI1\|KCNA5\|KIT\|OSR1\|SOD2\|TGFB2 |
| GO:0048568 | embryonic organ development | 6.22 × 10^−05^ | 1.43 × 10^−02^ | 7 | ACVR1\|BMP5\|GLI1\|KIT\|OSR1\|SLC44A4\|TGFB2 |
| GO:0051716 | cellular response to stimulus | 6.69 × 10^−05^ | 1.51 × 10^−02^ | 29 | ACVR1\|ACVR1C\|ACVR2A\|AMHR2\|ARHGAP28\|ATOH8\|BAMBI\|BMP5\|BTC\|CHRD\|CLEC5A\|EDNRB\|EPHA7\|GLI1\|IL1RL1\|ILDR1\|KCNA5\|KIT\|OR2M5\|OR4C12\|OR5F1\|OSR1\|PDE1C\|PDE7B\|PRKCD\|S100A14\|SCARA5\|SOD2\|TGFB2 |
| GO:0040012 | regulation of locomotion | 7.47 × 10^−05^ | 1.65 × 10^−02^ | 10 | ACVR1\|ACVR1C\|ATOH8\|BMP5\|BTC\|CHRD\|KIT\|S100A14\|SOD2\|TGFB2 |
| GO:0003203 | endocardial cushion morphogenesis | 7.81 × 10^−05^ | 1.68 × 10^−02^ | 3 | ACVR1\|BMP5\|TGFB2 |
| GO:0051270 | regulation of cellular component movement | 8.21 × 10^−05^ | 1.68 × 10^−02^ | 10 | ACVR1\|ACVR1C\|ATOH8\|BMP5\|BTC\|CHRD\|KIT\|S100A14\|SOD2\|TGFB2 |
| GO:0090100 | positive regulation of transmembrane receptor protein serine/threonine kinase signaling pathway | 1.04 × 10^−04^ | 2.09 × 10^−02^ | 4 | ACVR1\|ACVR2A\|BMP5\|TGFB2 |
| GO:0051240 | positive regulation of multicellular organismal process | 1.24 × 10^−04^ | 2.39 × 10^−02^ | 13 | ACVR1\|ACVR2A\|ATOH8\|BAMBI\|BMP5\|CLEC5A\|EDNRB\|GLI1\|IL1RL1\|KIT\|OSR1\|SOD2\|TGFB2 |
| GO:0050678 | regulation of epithelial cell proliferation | 1.27 × 10^−04^ | 2.41 × 10^−02^ | 6 | ATOH8\|BMP5\|EDNRB\|GLI1\|OSR1\|TGFB2 |
| GO:0030334 | regulation of cell migration | 1.31 × 10^−04^ | 2.41 × 10^−02^ | 9 | ACVR1\|ACVR1C\|ATOH8\|BMP5\|CHRD\|KIT\|S100A14\|SOD2\|TGFB2 |
| GO:0051239 | regulation of multicellular organismal process | 1.34 × 10^−04^ | 2.43 × 10^−02^ | 18 | ACVR1\|ACVR1C\|ACVR2A\|ATOH8\|BAMBI\|BMP5\|CHRD\|CLEC5A\|EDNRB\|EPHA7\|GLI1\|IL1RL1\|KCNA5\|KIT\|OSR1\|PRKCD\|SOD2\|TGFB2 |
| GO:0003006 | developmental process involved in reproduction | 1.42 × 10^−04^ | 2.50 × 10^−02^ | 8 | ACVR1\|ACVR2A\|AMHR2\|BMP5\|GLI1\|KIT\|OSR1\|TGFB2 |
| GO:0003197 | endocardial cushion development | 1.45 × 10^−04^ | 2.50 × 10^−02^ | 3 | ACVR1\|BMP5\|TGFB2 |
| GO:0043583 | ear development | 1.64 × 10^−04^ | 2.63 × 10^−02^ | 5 | BMP5\|FREM2\|OSR1\|SLC44A4\|TGFB2 |
| GO:0048513 | animal organ development | 1.65 × 10^−04^ | 2.63 × 10^−02^ | 18 | ACVR1\|ACVR2A\|AMHR2\|B3GNT5\|BMP5\|CHRD\|CLEC5A\|COL21A1\|EDNRB\|EPHA7\|FREM2\|GLI1\|KIT\|KRTAP12−4\|OSR1\|PCDH18\|SLC44A4\|TGFB2 |
| GO:0060485 | mesenchyme development | 1.67 × 10^−04^ | 2.63 × 10^−02^ | 5 | ACVR1\|BMP5\|EDNRB\|OSR1\|TGFB2 |
| GO:0042221 | response to chemical | 1.79 × 10^−04^ | 2.77 × 10^−02^ | 22 | ACVR1\|ACVR1C\|ACVR2A\|AMHR2\|BAMBI\|BMP5\|CHRD\|EDNRB\|EPHA7\|IL1RL1\|ILDR1\|KCNA5\|KIT\|OR2M5\|OR4C12\|OR5F1\|OSR1\|PRKCD\|S100A14\|SLC44A4\|SOD2\|TGFB2 |
| GO:0043067 | regulation of programmed cell death | 2.07 × 10^−04^ | 3.04 × 10^−02^ | 12 | ACVR1\|ACVR1C\|BMP5\|BTC\|CLEC5A\|EDNRB\|EPHA7\|KIT\|OSR1\|PRKCD\|SOD2\|TGFB2 |
| GO:0043069 | negative regulation of programmed cell death | 2.12 × 10^−04^ | 3.05 × 10^−02^ | 9 | ACVR1\|BMP5\|BTC\|CLEC5A\|EDNRB\|KIT\|OSR1\|PRKCD\|SOD2 |
| GO:0071310 | cellular response to organic substance | 2.33 × 10^−04^ | 3.29 × 10^−02^ | 15 | ACVR1\|ACVR1C\|ACVR2A\|AMHR2\|BAMBI\|BMP5\|CHRD\|EDNRB\|IL1RL1\|ILDR1\|KIT\|OSR1\|PRKCD\|SOD2\|TGFB2 |
| GO:0051094 | positive regulation of developmental process | 2.36 × 10^−04^ | 3.29 × 10^−02^ | 11 | ACVR1\|ACVR2A\|ATOH8\|BAMBI\|BMP5\|BTC\|GLI1\|KIT\|OSR1\|SOD2\|TGFB2 |
| GO:0010033 | response to organic substance | 2.62 × 10^−04^ | 3.53 × 10^−02^ | 17 | ACVR1\|ACVR1C\|ACVR2A\|AMHR2\|BAMBI\|BMP5\|CHRD\|EDNRB\|IL1RL1\|ILDR1\|KCNA5\|KIT\|OSR1\|PRKCD\|S100A14\|SOD2\|TGFB2 |
| GO:0009653 | anatomical structure morphogenesis | 2.86 × 10^−04^ | 3.72 × 10^−02^ | 14 | ACVR1\|ACVR2A\|AMHR2\|ATOH8\|BMP5\|CHRD\|COL21A1\|EPHA7\|FREM2\|GLI1\|GSC2\|OSR1\|SLC44A4\|TGFB2 |
| GO:0060395 | SMAD protein signal transduction | 3.19 × 10^−04^ | 4.04 × 10^−02^ | 3 | ATOH8\|BMP5\|TGFB2 |
| GO:0045597 | positive regulation of cell differentiation | 3.47 × 10^−04^ | 4.34 × 10^−02^ | 9 | ACVR1\|ACVR2A\|ATOH8\|BAMBI\|BMP5\|BTC\|KIT\|SOD2\|TGFB2 |
| GO:0032502 | developmental process | 3.81 × 10^−04^ | 4.54 × 10^−02^ | 25 | ACVR1\|ACVR1C\|ACVR2A\|AMHR2\|ATOH8\|B3GNT5\|BMP5\|CHRD\|CLEC5A\|COL10A1\|COL21A1\|EDNRB\|EPHA7\|FREM2\|GLI1\|GSC2\|KIT\|KRTAP12−4\|MARVELD2\|OSR1\|PCDH18\|PRKCD\|SLC44A4\|SOD2\|TGFB2 |
| GO:0035148 | tube formation | 3.97 × 10^−04^ | 4.66 × 10^−02^ | 4 | ATOH8\|BMP5\|OSR1\|TGFB2 |
| GO:0010941 | regulation of cell death | 4.12 × 10^−04^ | 4.78 × 10^−02^ | 12 | ACVR1\|ACVR1C\|BMP5\|BTC\|CLEC5A\|EDNRB\|EPHA7\|KIT\|OSR1\|PRKCD\|SOD2\|TGFB2 |
| GO:0060548 | negative regulation of cell death | 4.22 × 10^−04^ | 4.82 × 10^−02^ | 9 | ACVR1\|BMP5\|BTC\|CLEC5A\|EDNRB\|KIT\|OSR1\|PRKCD\|SOD2 |
| GO:0071495 | cellular response to endogenous stimulus | 4.44 × 10^−04^ | 4.99 × 10^−02^ | 10 | ACVR1\|ACVR1C\|ACVR2A\|AMHR2\|BAMBI\|BMP5\|CHRD\|KIT\|PRKCD\|TGFB2 |

**Figure S1:** Copy number alteration frequency of each gene of the BMP5 cluster from GeneMANIA and STRING database. A total of 17815 patients / 18300 samples in 30 studies were queried. Queried genes were altered in 6751 (38%) of queried patients in 6825 (37%) of queried samples. The alterations include Inframe mutations−unknown significance (Brown), Missense mutations−putative driver (Deep green), missense mutations−unknown significance (Green), truncating mutations−putative driver (Black), truncating mutations−unknown significance (Grey), fusions (Violet), amplifications (Red), deep deletions (Deep Blue), no alterations (Light Grey) and not profiled cases (white).


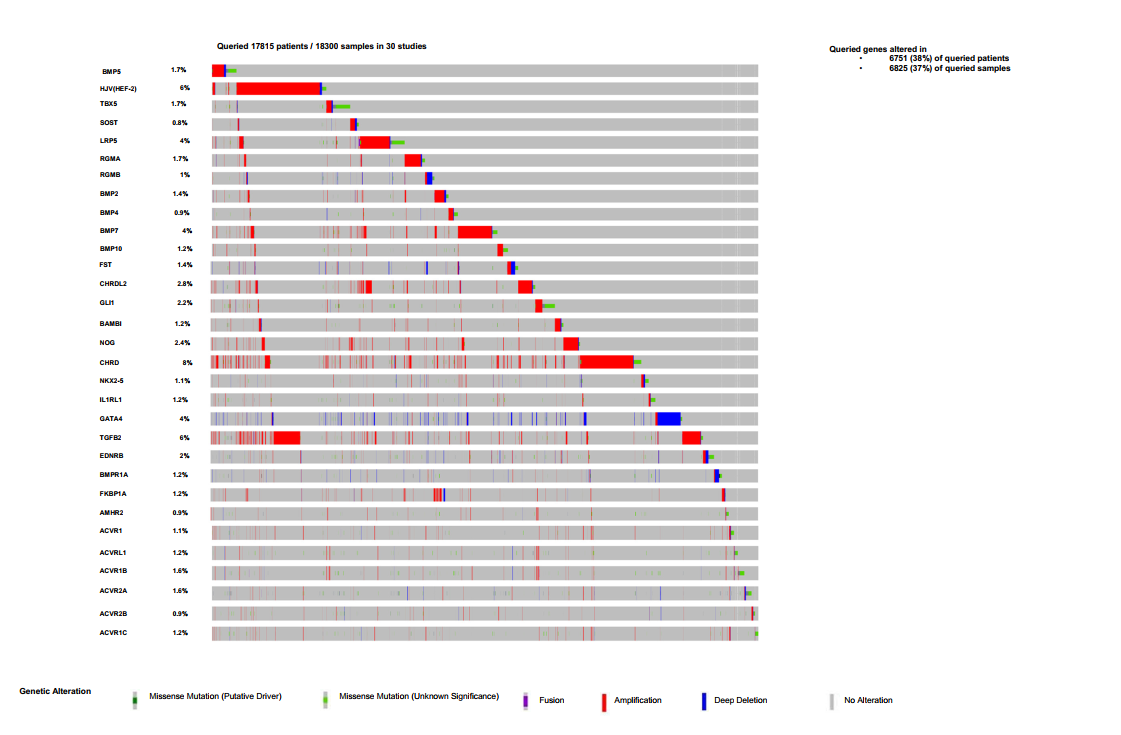


**S2 Figure:** Pathway analysis of *BMP5* using PANTHER pathway tool. **A**. A total of 49 biological processes were reported and distributed in eight categories. **B**. A total of 26 cellular components were reported and distributed in four categories. **C**. A total of 34 molecular functions were reported and distributed in seven categories. **p* <0.05.


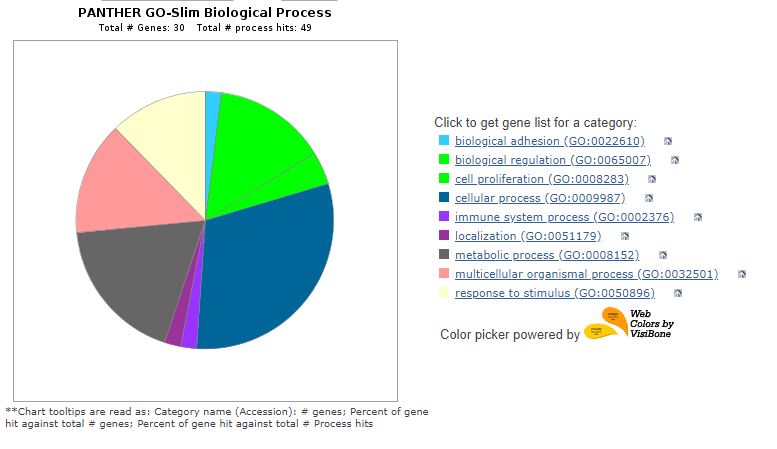


A


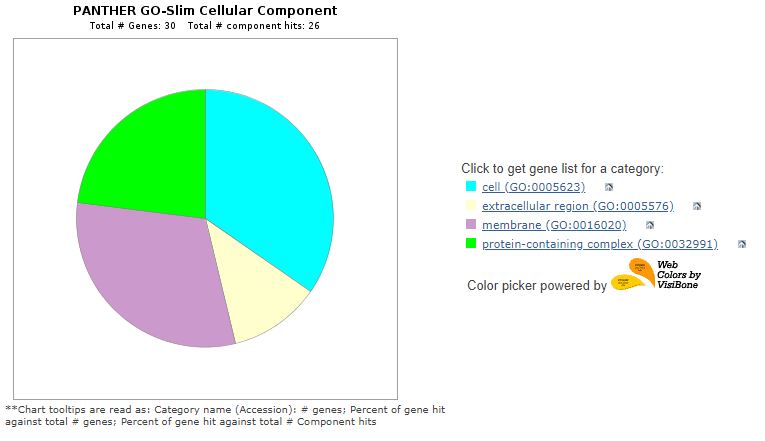


B


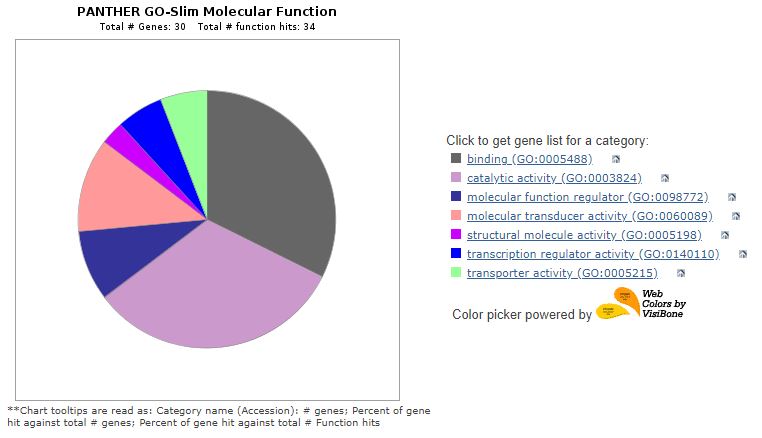


C
